# A preliminary investigation of anticholinesterase activity of some Iranian medicinal plants commonly used in traditional medicine

**DOI:** 10.1186/2008-2231-22-17

**Published:** 2014-01-08

**Authors:** Seyed Behzad Jazayeri, Arash Amanlou, Naghmeh Ghanadian, Parvin Pasalar, Massoud Amanlou

**Affiliations:** 1 Students’ Scientific Research Center, Tehran University of Medical Sciences, Tehran, Iran; 2 Department of Medicinal Chemistry, Faculty of Pharmacy and Medicinal Plants Research Center, Tehran University of Medical Sciences, Tehran, Iran; 3 Department of Clinical Biochemistry, Faculty of Medicine, Tehran University of Medical Sciences, Tehran, Iran

**Keywords:** Plant extracts, Acetylcholinesterase inhibitor, Alzheimer’s disease, Camellia sinensis, Citrus aurantifolia

## Abstract

**Background:**

The aim of this study was to evaluate acetylcholinesterase inhibitory activity of some commonly used herbal medicine in Iran to introduce a new source for management of Alzheimer’s disease. A total of 18 aqueous-methanolic extract (1:1; v/v) from the following plants: *Brassica alba*, *Brassica nigra*, *Camellia sinensis*, *Cinchona officinalis*, *Citrus aurantifolia*, *Citrus x aurantium*, *Ferula assafoetida*, *Humulus lupulus*, *Juglans regia*, *Juniperus sabina*, *Myristica fragrans*, *Pelargonium graveolens*, *Pistacia vera*, *Punica granatum*, *Rheum officinale*, *Rosa damascena*, *Salix alba*, *and Zizyphus vulgaris* were prepared and screened for their acetylcholinesterase inhibitory activity using *in vitro* Ellman spectrophotometric method.

**Results:**

According to the obtained results, the order of inhibitory activity (IC_50_ values, μg /ml) of extracts from highest to the lowest was: *C. sinensis* (5.96), *C. aurantifolia* (19.57), *Z. vulgaris* (24.37), *B. nigra* (84.30) and *R. damascena* (93.1).

**Conclusions:**

The results indicated and confirmed the traditional use of these herbs for management of central nervous system disorders. *C. sinensis* showed the highest activity in inhibition of acetylcholinesterase. However, further investigations on identification of active components in the extracts are needed.

## Finding

Alzheimer’s disease (AD) is a an age related neurodegenerative disorder with clinical characteristic and pathological features associated with loss of neurons in certain brain areas leading to impairment of memory, cognitive dysfunction, behavioral disturbances, deficits in activities of daily living, which eventually leads to death [1-3]. In 2010, approximately 35 million people worldwide were suffering from AD and this number is believed to reach 65.7 million by 2030 [4].

Although the underlying pathophysiological mechanisms are not clear, AD is firmly associated with impairment in cholinergic pathway, which results in decreased level of acetylcholine in certain areas of brain [1,2,5,6].

The management of AD focuses on slowing disease progression, symptomatic treatment, maintaining functional status and improving quality of life, and decreasing caregiver stress [5]. Acetylcholine, a neurotransmitter, which is hydrolyzed by acetylcholinesterase (AChE) and butyrylcholinesterase (BuChE) is considered to play an important role in the pathology of AD [1,2,7].

The treatment of AD has progressed and shifted since the late 1970s to a transmitter replacement strategy. Elevation of acetylcholine levels in brain through the use of AChE inhibitors has been accepted as the most effective treatment strategy against AD [3,8]. Therefore, AChE and/or BuChE inhibitors have become the drug of choice in management of AD [9]. Several AChE inhibitors named as “cognitive enhancers “are being investigated for the symptomatic treatment of Alzheimer’s disease but few have been approved by the Food and Drug Administration in the United States [2,8,10,11]. The few drugs that have received regulatory approval to this date include: donepezil, rivastigmine and galantamine, all three working through increasing the concentration of acetylcholine at the neurotransmitter sites or acts by regulating activity at nicotinic receptors [2,5].

However, studies investigating the use of medications for AD have not been consistently supportive [12-17]. Various side effects of medications reported in clinical trials include: nausea, vomiting, diarrhea, syncope and bradycardia. Consequently, a need for development and utilization of alternative anticholinesterase compounds with fewer side effects leads to investigation on plants as a possible source of treatment [18-26]. Plants have been used since antiquity in the treatment of various diseases including cognitive disorders, such as AD.

Considering the importance of plant-driven compounds in drug discovery, the present study was undertaken to evaluate the anticholinesterase activity of a number of selected medicinal plants with various ethnobotanical uses, aiming to discover new candidates for anticholinesterase activity to be used in management of AD.

## Material and methods

### Plant materials

Eighteen medicinal plants, which are listed in Table 1 were chosen randomly from local herbal market in September 2010, Tehran and identified by Dr. Faraz Mojab. Voucher specimens were kept in the Herbarium of Faculty of Pharmacy, Shahid Beheshti University of Medical Sciences, Tehran, Iran.

**Table 1:** **List of medicinal plants**, **traditional use and anticholinesterase activity against electric eel acetylcholinesterase**

**Botanical name**	**Plant family**	**Parts tested**	**Common name**	Traditional uses^*^	**Percent of inhibition(IC**_**50**_**); μg/ml**
*Brassica alba*	Brassicaceae	Seed	White mustard	Chest congestion, bronchitis, swollen joints, rheumatism	84.3 ± 1.36
*Brassica nigra*	Brassicaceae	Seed	Mustard	Common cold, painful joints and muscles, arthritis	135.0 ± 5.91
*Camellia sinensis*	Theaceae	Leaf	Tea	Relieve mental and physical fatigue, antioxidant	5.96 + 0.73
*Cinchona officinalis*	Rubiaceae	Bark	Quina-quina	Muscle relaxant, anti-malaria, palpitations, anemia, diarrhea	187.6 ± 4.25
*Citrus aurantifolia*	Rutaceae	Fruit	Key lime	Alertness, anti-flatulent, uplifting and cheering the spirit, common cold, anti- inflammatory	19.57 ± 2.66
*Citrus x aurantium*	Rutaceae	Flower	Bitter orange	Digestive aid and sedative, regulating fertility	226.1 ± 7.41
*Ferula assafoetida*	Apiaceae	Gum	Asafoetida	Anti-inflammatory, antispasmodic, cancer, colitis	281.3 ± 5.23
*Humulus lupulus*	Cannabaceae	Flower	Hop	Sedative, stimulate digestion, insomnia, anti-anxiety	369.6 ± 9.82
*Juglans regia*	Juglandaceae	External shell	Walnut	Antimicrobial, antihelminthic, astringent, keratolytic	647.5 ± 8.61
*Juniperus sabina*	Cupressaceae	Fruit	Savin	Antifertility, antioxidant, anti-inflammatory	379.9 ± 9.38
*Myristica fragrans*	Myristicaceae	Seed	Nutmeg	Diarrhea, mouth sores, and insomnia	1024. ±11.02
*Pelargonium graveolens*	Geraniaceae	Areal parts	Rose geranium	Reducing stress and tension, easing pain,	196.9 ± 7.25
*Pistacia vera*	Anacardiaceae	Hull	Pistachio	Antiviral, antifungal, antiprotozoal, anti-inflammatory activity	204.1 ± 6.33
*Punica granatum*	Lythraceae	Fruit	Pomegranate	Gastrointestinal disorders, astringent, diarrhea, oral aphthous, hematopoiesis	408.2 ± 5.72
*Rheum officinale*	Polygonaceae	Root	Rhubarb	Astringent, antibacterial, laxative	341.7 ± 3.88
*Rosa damascena*	Rosaceae	Flower	Damask rose	Cardiovascular stimulant, mild laxative, anti-inflammatory, cough suppressant	93.1 ± 2.88
*Salix alba*	Salicaceae	Bark	White willow	Chronic diarrhea, reduction of fevers, menstrual irregularities	989.1 ± 4.29
*Zizyphus vulgaris*	Rhamnaceae	Fruit	Jujube	Aphrodisiac, hypnotic-sedative, anxiolytic, anti-inflammatory	24.37 ± 2.33

* Traditional uses mentioned in this table are obtained from Avicenna’s cannon of medicine, herbal shops instructions, and general usages in traditional medicine of Iran.

### Chemicals

Acetylthiocholine iodide (ATCI), 5,5’–dithio-bis-2-nitrobenzoic acid (DTNB), bovine serum albumin (BSA) and electric eel AChE (AChE; EC 3.1.17; lyophilized, 500 U/vial solid, 65U/mg) were purchased from Sigma (St. Louis, MO). Physostigmine was used as the standard drug. Buffers and other chemical were of extra pure analytical grade. The following buffers were used: Buffer A: 50 mM Tris–HCl, pH 8, containing 0.1% BSA; Buffer B: 50 mM Tris- HCl, pH 8 containing 0.1 M NaCl, 0.02 M MgCl2 × 6H2O.

### Preparation of extracts

Each plant sample was individually powdered and 1 g of each sample was extracted by maceration method under shaking at room temperature with aqueous methanol (20 mL; 1:1 v/v) for 24 h. After filtration, organic layer was distilled under reduced pressure at 25°C and then freeze-dried to dryness. The crude extracts were stored at -20°C until analysis. Determination of fifty percent inhibitory concentrations (IC_50_) at 100 μg/mL dissolved in aqueous methanol was accurately defined.

### Anticholinesterase inhibitory activity

Ellman’s method was employed for determination of AChE inhibitory activity [26-28]. Acetylthiocholine was used as a substrate and hydrolysis of acetylthiocholine was determined by monitoring the formation of the yellow 5-thio-2-nitrobenzoate anion as a result of the reaction with 5,5’–dithio-bis-2-nitrobenzoic acid with thiocholine, catalyzed by enzymes at a wavelength of 412 nm.

Briefly, 25 μl of 15 mM ATCI, (43 mg/10 mL in Millipore water), 125 μl of 3 mM DTNB, (11.9 mg/10 mL buffer B), 50 μl of buffer A and 20 μl of plant extract at concentration of 100 μ g/ml were added to 96 well plates and the absorbance was measured at 412 nm every 13 s for five times. After adding 25 μl of 0.22 U/ml enzyme, (0.34 mg AChE dissolved in 100 mL buffer A), the absorbance was read again every 13 seconds for five times. The absorbance was measured using a Synergy H4 Hybrid Multi-Mode Microplate Reader (BioTek Instruments Inc., United States). Percentage of inhibition was calculated by comparing rates of the sample with the blank (aqueous methanol), control samples contained all components except the tested extract. Physostigmine was used as positive control. Then, the mean ofthree measurements for each concentration was determined (n = 3). Inhibitory concentration (IC_50_ value) was calculated according to Michaelis–Menten model by using EZ-Fit. Enzyme Inhibition Kinetic Analysis program (EZ-Fit: Enzyme Kinetics Software, Perrella Scientific Inc., Amshert, USA).

### Statistical method

The assays were conducted in triplicate (n = 3) and after calculating the mean ± SD, the results were compared using Student’s *t*-*test*. A *P* value of less than 0.05 was considered significant.

## Results and discussion

Eighteen plant species belonging to 16 plant families (*Anacardiaceae*, *Apiaceae*, *Brassicaceae*, *Cannabaceae*, *Cupressaceae*, *Geraniaceae*, *Juglandaceae*, *Lythraceae*, *Myristicaceae*, *Polygonaceae*, *Rhamnaceae*, *Rosaceae*, *Rubiaceae*, *Rutaceae*, *Salicaceae*, and *Theaceae*) were obtained from Tehran local herbal market and a total of 18 extracts were screened for AChE inhibitory activity using Ellman’s spectrophotometric method in 96-well microplate. Table 1 gives the names of the plants investigated, their families, their traditional uses, and acetylcholinesterase inhibitory concentration (IC_50_) respectively. IC_50_ of physostigmine (positive control) was estimated 0.093 μM against AChE. At the test extracts concentration (100 μg/ml), physostigmine showed complete inhibition of enzyme activity.

### Camellia sinensis

Results showed that AChE inhibitory concentration (IC_50_) of leaves of *C. sinensis* (5.96 μg/ml) was less than the inhibitory concentration (IC_50_) of the other tested extracts (Table 1). An inhibitory activity has been reported for green leaves of *C. sinensis* previously [29]. The occurrence of flavonoid saponins, polyphenols, and catechins is well documented in different preparations of *C. sinensis*. Quantitative concentrations of these compounds in tea infusion might be different due to impact of preparation method, cultivar, and agricultural divergences, which might explain the observed difference in AChE inhibitory activity between different types of *C. sinensis*[30].

### Citrus aurantifolia

In this study *C. aurantifolia* presented an IC_50_ inhibitory effect of AChE in concentration of 19.57 (μ g/ml). Report by Chaiyana and Okonogi [31] revealed inhibition of cholinesterase by essential oil of leaf and fruit peel of *C. aurantifolia*. Phytochemical investigations of *C. aurantifolia* revealed the occurrence of limonene, l-camphor, citronellol, o-cymene and 1,8-cineole as the major constituents [31]; A group of compounds reported to have AChE inhibitory activity [31]. Essential oils from same family have been reported to posses promising AChE inhibitory activity [32]. However, there is no AChE inhibitory activity in blooms of C. *aurantifolia*.

### Zizyphus vulgaris

The extract from *Z. vulgaris* fruit showed moderate AChE inhibitory activity (IC_50_ = 24.37 μg/ml) in this study, which is similar to the results of previous studies [33,34]. The AChE inhibitory activity of *Z. vulgaris* can be explained by the presence of alkaloids, saponins, and flavonoids in the extract [33].

### Rosa damascena

We found AChE inhibitor activity from *R. damascena* floret extract (IC_50_ = 93.10 μg/ml) that is not in supported in previous investigations [25]. The *R. damascena* floret jam is used traditionally as a sweetener to tea to increase memory and wellness.

### Brassica nigra

There is limited data on seeds of *B. nigra*. This study shows that seeds of *B. nigra* present AChE inhibitory activity with an IC_50_ of 135.0 (IC_50_ = 93.10 μg/ml). This finding suggests a moderate inhibitory activity for *B. nigra* seeds. Though further investigation in active components of the extract is needed.

The promising finding of the study shows that most of the plant-extracts screened in this study had some degrees of inhibitory activity against AChE (Table 1), but five species showed the most active inhibitory property (*C. sinensis*, *C. aurantifolia*, *Z. vulgaris*, *B. nigra*, *and R. damascena*) and had lowest inhibitory concentration below 100 μg/ml ranging between 5.96 to 93.10 against electric eel AChE. The extract of the herbs alone or in combination of other herbal productions such as essential oil could be considered in herbal remedies of AD management. Since the most strong synthetic or natural product driven AChE inhibitors are known to contain nitrogen, the promising activity of reported medicinal plants could be due to their high alkaloidal contents [35-40]. Alkaloids are the major compounds isolated from plants and show inhibitory activity for AChE [35-40] but only one out of each five most potent species contains alkaloids.

The search performed using Chemical Abstracts, Biological Abstracts and Scopus database shows that AChE inhibitory activity is not only limited to alkaloids but also other compounds such as flavonoid, coumarins and essential oils are reported to have AChE inhibitory activity [35-41]. The finding of this study shows that the four most active herbal extracts contained not only nitorgenic compound such as alkaloid but also these extracts also contain rich components of saponin, flavonoid, and essential oils [29-34]. The new area of interest in research involves both AChE inhibitors and BuChE inhibitors after recognition of BuChE activity in hippocampus of patients with AD [33,42,43]. Recent studies have work on dual inhibitors of AChE and BuChE [42,43]. In these studies synthesized chemical compounds have shown promising inhibitory effect on both AChE and BuChE [42-44]. However, the present study was limited to the screening of AChE inhibitory properties of selected plants. Thus, further studies should focus on BuChE inhibitory activity of herbal products of the plants used in this study and other potent plants.

## Conclusions

A primary screening process was run to investigate AChE inhibitory properties of medicinal plants of Iran. The primary findings of this study suggest that all herbs used in this study exhibited some degree of AChE inhibitory properties. Among the selected plants of this report *C. sinensis* had the most active components with inhibitory properties on AChE. Further researches should investigate more on the chemical composition and mechanism of actions of these herbal extract including *in vitro* and *in vivo* studies.

## Abbreviations

AChE: Acetylcholinesterase; AD: Alzheimer’s disease.; BuChE: Butyrylcholinesterase.

## Competing interests

The authors declare that they have no competing interests.

## Authors’ contributions

All authors contributed to the concept and design, making and analysis of data, drafting, revising and final approval. MA and PP are responsible for the study registration. SBJ, AA and NG carried out plant extraction and enzymatic tests and drafted manuscript. SBJ, PP and MA participated in collection and/or assembly of data, data analysis, interpretation and manuscript writing. All authors read and approved the final manuscript.
